# Response on “commentary on “using resonance synchronous spectroscopy to characterize the reactivity and electrophilicity of biologically relevant sulfane sulfur”. Evidence that the methodology is inadequate because it only measures unspecific light scattering”. The evidence is incorrect

**DOI:** 10.1016/j.redox.2019.101312

**Published:** 2019-09-03

**Authors:** Huaiwei Liu, Qingda Wang, Huanjie Li, Luying Xun

**Affiliations:** a State Key Laboratory of Microbial Technology, Shandong University, 72 Binhai Road, Qingdao, 266237, People's Republic of China; b School of Medicine, Shandong University, 44 Wenhua Xi Road, Jinan, 250012, People's Republic of China; c School of Molecular Biosciences, Washington State University, Pullman, WA, 99164-7520, USA

Sulfane sulfur, including HS_n_H and RS_n_H, n ≥ 2; RS_n_R, n ≥ 3, contains zero-valent sulfur (S^0^). It is a newly discovered cellular component with important physiological functions, including redox homeostasis maintenance and signaling [1]. Due to the diversity of sulfane sulfur species, their chemical properties are still largely unknown. We recently discovered that biologically relevant sulfane sulfur species display strong optical signals when analyzed by resonance synchronous spectroscopy (RS_2_), in which excitation and emission wavelengths are essentially identical [2]. We reported that several sulfane sulfur species, including inorganic polysulfide (H_2_S_n_, HS_n_^−^, and S_n_^2−^), glutathioine persulfide (GSSH), protein persulfide, and organic polysulfide (RS_n_H, n ≥ 2 and RS_n_R, n ≥ 3) have RS_2_ signals, which are affected by pH if the sulfane sulfur species undergo protonation and deprotonation [2].

After our publication, Cuevasanta et al. published a commentary, claiming that RS_2_ does not measure soluble sulfane sulfur but elemental sulfur particles derived from soluble sulfane sulfur [3]. However, they only did two inappropriate experiments without any quantification, leading to a wrong conclusion that our method “only measures unspecific light scattering”.

For the first experiment, they showed colloidal sulfur, prepared by vortexing sulfur powder into water, also displayed RS_2_ signals, suggesting that our reported RS_2_ of sulfane sulfur is due to light scattering of sulfur particles [3]. We performed a similar experiment, diluting inorganic polysulfide (26 mM stock in an alkaline solution [4]) to 1.5 μM in 50 mM Tris buffer (pH 7.4) for RS_2_ analysis. We then prepared colloidal sulfur by vortexing sulfur powder in the same buffer [3]. The suspension was allowed to settle for 1 hour, and the supernatant was diluted with equal volume of the same buffer before RS_2_ analysis. The data are presented as R_2_S_2_ in which the buffer's RS_2_ is corrected [5]. This correction is necessary, as Tris buffer has background RS_2_ signals (Fig. 1A in [2]). Cuevasanta et al. used water instead of a buffer and did not use R_2_S_2_ [3]. The R_2_S_2_ spectra are similar but different when compared via overlaying, as the colloidal sulfur spectrum is red-shifted (Fig. 1, black vs. blue). The RS_2_ signal of the polysulfide solution was unstable and mostly disappeared after 5 min (Fig. 1, black, red, green, and grey), while that of colloidal sulfur was stable, showing no reduction within 30 min (Fig. 2). Thus, the RS_2_ signals of polysulfide and colloidal sulfur are different.

**Fig. 1:**
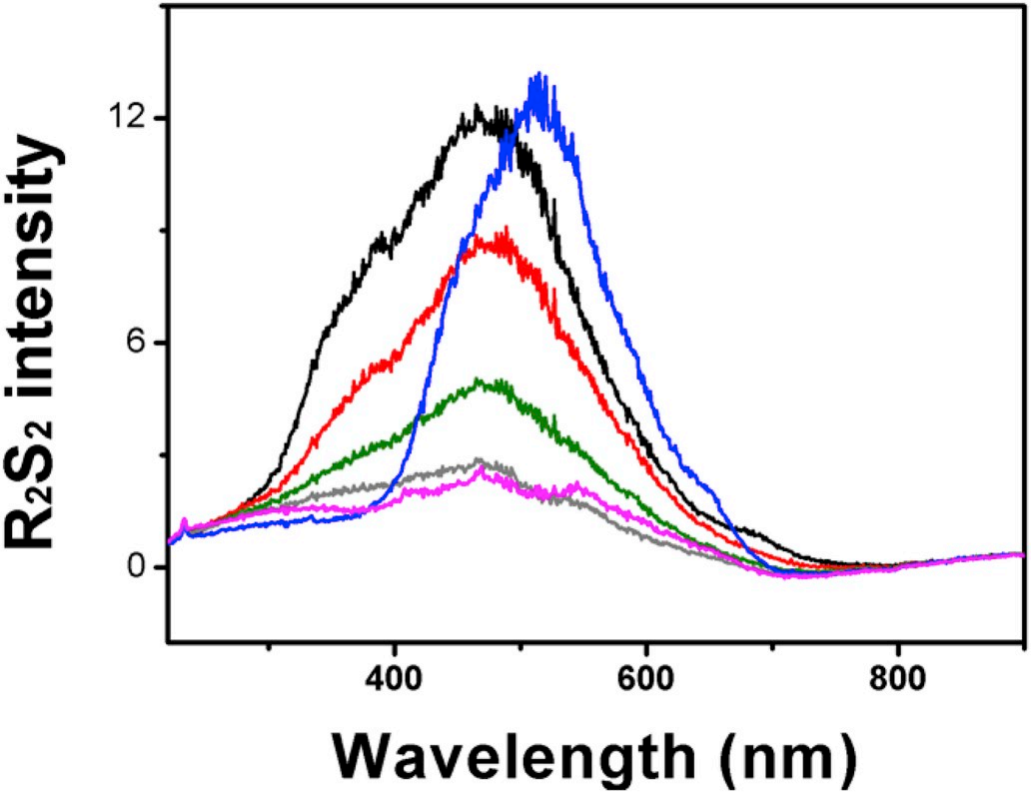
**R**_**2**_**S**_**2**_**spectra of polysulfide and colloidal sulfur. Black and red curves:** Polysulfide stock with sulfide in alkaline solution under anaerobic conditions was diluted to 1.5 μM in 50 mM Tris buffer (pH 7.4). **Black**, immediately; **red**, after 1 min; **green**, after 3 min, **grey**, after 5 min. **Blue curve:** Colloidal sulfur was prepared in the Tris buffer by vortexing. The colloidal sample was diluted with equal volume of the same buffer before RS_2_ analysis. **Pink curve:** Elemental sulfur was dissolved in acetone and diluted to 1.5 μM in the Tris buffer. R_2_S_2_ was obtained by correcting the RS_2_ signal of the buffer [5]. (For interpretation of the references to colour in this figure legend, the reader is referred to the web version of this article.)

**Fig. 2:**
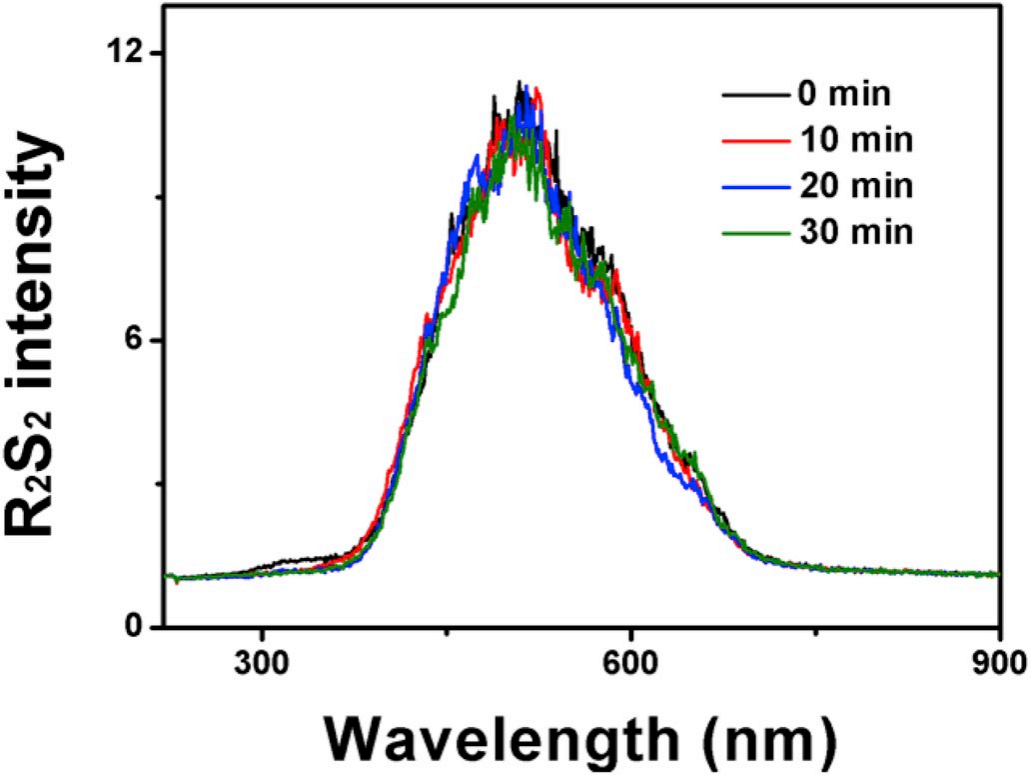
The RS_2_ of the colloidal sulfur solution (Fig. 1 legend) was stable within 30 min. (For interpretation of the references to colour in this figure legend, the reader is referred to the web version of this article.)

We have showed the presence of elemental sulfur S_8_ in inorganic polysulfide at neutral pH [2]. Polysulfide stock is prepared in alkaline solutions with sulfide in access under anaerobic conditions [4], and it is mainly present as long chain polysulfide species [2]. When the stock was diluted to 1.5 μM in 50 mM Tris buffer (pH 7.4), S_8_ could form at the relative neutral pH [2]. To test whether S_8_ was mainly responsible for RS_2_, we dissolved elemental sulfur in acetone (15 mM) and diluted it to 1.5 μM in 50 mM Tris buffer (pH 7.4). The R_2_S_2_ signal of S_8_ was much weaker, about 4.6-fold lower than that of 1.5 μM inorganic polysulfide (Fig. 1, pink). Thus, when inorganic polysulfide is diluted in 50 mM Tris buffer (pH 7.4), the initial RS_2_ signal is mainly from the polysulfide. The rapid loss of the signal is likely due to the conversion to S_8_ or the oxidation by O_2_. The produced S_8_ should aggregate into fine particles similar to that of S_8_, obtained via diluting sulfur stock in acetone into the same buffer; both should display reduced RS_2_ signals likely because of scattering and the sulfane sulfur property of S_8_ (Fig. 1), as RS_2_ is often used to analyzed aggregates of dye molecules [5,6].

For the second experiment, Cuevasanta et al. used 1 mM H_2_O_2_ to oxidize 1 mM H_2_S and claimed that the reaction also produced the reported signal [3]. They showed that the obtained signal was from small particles via light scattering. However, they did not show how long it took to generate the signal and how much sulfur particles were generated from 1 mM H_2_S. We repeated their experiment and could not detect the signal within 30 min. This is likely due to the high concentrations of H_2_O_2_ used in their experiment. Since H_2_O_2_ reacts with H_2_S at a much slower rate (0.46 M^−1^s^−1^, the 2^nd^ rate constant [2]) than with sulfane sulfur (23.76 M^−1^s^−1^ for GSSH reacting with H_2_O_2_ [2]), the produced polysulfide is not accumulated but rapidly oxidized by H_2_O_2_. The method cannot be used to prepare inorganic polysulfide, which is unstable even without H_2_O_2_ (Fig. 1). They misused our kinetic assay [3]. We used excess sulfide to react with 50 μM H_2_O_2_ and monitored polysulfide production by using RS_2_; we only used the data from the first 3 min to obtain the initial rate, from which the rate constant was calculated [2].

The R_2_S_2_ spectrum of GSSH at pH 6 (Fig. 2A of original paper [2]) has a maximum around 650 nm. In comparison, the R_2_S_2_ spectrum of colloidal sulfur in 50 mM phosphate buffer (pH 6) is essentially the same as that in 50 mM Tris buffer, pH 7.4, significantly different from that of GSSH [3]. As presented in Fig. 1C&D of our original paper [2], the R_2_S_2_ spectra of the commercially available Bis(methyl) trisulfide (CH_3_-SSS-CH_3_) and Bis[3-(triethoxysilyl)propyl] tetrasulfide are also different from that of colloidal sulfur. Thus, there is no evidence to suggest that these compounds decay to elemental sulfur during our assay. Cuevasanta et al. suggested that the reaction of these compounds with H_2_O_2_ or SSP4 (sulfane sulfur probe 4) as we tested is through colloidal sulfur without any supporting evidence [3]. We have not found any other reports suggesting that colloidal sulfur is an intermediate in these reactions.

RS_2_ is a data acquisition method by using a fluorometer. RS_2_ signals can be contributed by scattering, on-fluorescence, and possible Stokes’ shifted fluorescence [6]. Further, resonant Rayleigh scattering, caused by molecular polarity, may also contribute to RS_2_ [7,8]. The electrophilic property of sulfane sulfur could be polar when containing a thiosulfoxide bond [2]. We did not observe any fluorescence besides RS_2_ signals for all tested sulfane sulfur. However, a compound does not have to be fluorescent to give RS_2_ signals, as evidence by the RS_2_ spectrum of 50 mM Tris buffer (Fig. 1A in [2]). In short, the RS_2_ property of sulfane sulfur is unexpected, but is real.

## Funding

The work was financially supported by a grant from the National Key Research and Development Program of China (2016YFA0601103).
